# German federal-state-wide seroprevalence study of 1^st^ SARS-CoV-2 pandemic wave shows importance of long-term antibody test performance

**DOI:** 10.1038/s43856-022-00100-z

**Published:** 2022-05-18

**Authors:** Stefan Lohse, Anna Sternjakob-Marthaler, Paul Lagemann, Jakob Schöpe, Jürgen Rissland, Nastasja Seiwert, Thorsten Pfuhl, Alana Müllendorff, Laurent S. Kiefer, Markus Vogelgesang, Luca Vella, Katharina Denk, Julia Vicari, Anabel Zwick, Isabelle Lang, Gero Weber, Jürgen Geisel, Jörg Rech, Bernd Schnabel, Gunter Hauptmann, Bernd Holleczek, Heinrich Scheiblauer, Stefan Wagenpfeil, Sigrun Smola

**Affiliations:** 1 Institute of Virology, Saarland University Medical Center, 66421 Homburg, Germany; 2 Institute for Medical Biometry, Epidemiology and Medical Informatics, Saarland University Medical Center, 66421 Homburg, Germany; 3 Physical Geography and Environmental Research, Saarland University, 66125 Saarbrücken, Germany; 4 Central Laboratory, Saarland University Hospital, 66421 Homburg, Germany; 5 Ministry of Health, Social Affairs, Women and the Family, 66119 Saarbrücken, Germany; 6 Kassenärztliche Vereinigung Saarland, 66113 Saarbrücken, Germany; 7 Saarland Cancer Registry, 66117 Saarbrücken, Germany; 8 Paul-Ehrlich-Institut, 63225 Langen, Germany; 9 Helmholtz Institute for Pharmaceutical Research Saarland (HIPS), Helmholtz Centre for Infection Research (HZI), Saarland University Campus, 66123 Saarbrücken, Germany

**Keywords:** Viral infection, Population screening

## Abstract

**Background:**

Reliable data on the adult SARS-CoV-2 infection fatality rate in Germany are still scarce. We performed a federal state-wide cross-sectional seroprevalence study named SaarCoPS, that is representative for the adult population including elderly individuals and nursing home residents in the Saarland.

**Methods:**

Serum was collected from 2940 adults via stationary or mobile teams during the 1^st^ pandemic wave steady state period. We selected an antibody test system with maximal specificity, also excluding seroreversion effects due to a high longitudinal test performance. For the calculations of infection and fatality rates, we accounted for the delays of seroconversion and death after infection.

**Results:**

Using a highly specific total antibody test detecting anti-SARS-CoV-2 responses over more than 180 days, we estimate an adult infection rate of 1.02% (95% CI: [0.64; 1.44]), an underreporting rate of 2.68-fold (95% CI: [1.68; 3.79]) and infection fatality rates of 2.09% (95% CI: (1.48; 3.32]) or 0.36% (95% CI: [0.25; 0.59]) in all adults including elderly individuals, or adults younger than 70 years, respectively.

**Conclusion:**

The study highlights the importance of study design and test performance for seroprevalence studies, particularly when seroprevalences are low. Our results provide a valuable baseline for evaluation of future pandemic dynamics and impact of public health measures on virus spread and human health in comparison to neighbouring countries such as Luxembourg or France.

## Plain language summary

To date, few reliable data are available on the proportion of adults who were infected with SARS-CoV-2 or the proportion who died from COVID-19 during the first pandemic wave in Germany. We tested a representative subset of adults in the Saarland region of Germany for antibodies, proteins circulating in the blood that are indicative of previous infection with SARS-CoV-2. We used three different antibody tests at different time points after the first pandemic wave and compared the results obtained using the different tests. Using the test with the best performance, we estimate nearly three fold of infections were previously unreported. We estimate 2.09% of adults who were infected with SARS-CoV-2 went on to die. This reduced to 0.36% for individuals under 70 years of age. Our data highlight the importance of selecting the right antibody test system with high longitudinal test performance for such studies.

## Abstract (web summary)

Lohse, Sternjakob-Marthaler et al. assess anti-SARS-CoV-2 antibody responses of adults in the Saarland region of Germany after the first pandemic wave. They calculate infection and fatality rates, demonstrating that SARS-Cov-2 infections during this period were underreported.

## Introduction

The pandemic due to SARS-CoV-2 (severe acute respiratory syndrome coronavirus 2) dominates human life, health systems and global economies for more than one year. On November 5, 2021, the WHO reported more than 247 million infections and 5 million deaths from Corona Virus Disease 2019 (COVID-19) worldwide (https://covid19.who.int/). To implement adequate measures, authorities need reliable data to evaluate the dynamics of viral spread, the effects of upcoming viral variants on human health, as well as the impact of vaccination. Severe cases requiring medical care and hospitalization, or fatal cases are recorded on a daily basis in developed countries. It can be assumed, however, that reported numbers of cases with mild or asymptomatic infections can vary grossly in populations depending on implemented test strategies and test frequencies. Accordingly, fatality rates calculated based on reported cases may vary widely depending on the number of unrecorded cases^1–5^.

Seroepidemiology providing information on infections retrospectively can help to overcome this gap. To uncover previously unrecorded infections as accurately as possible in seroepidemiological studies, a long-term longitudinal antibody detection capability along with a high test performance (specificity and sensitivity) is of utmost importance. Particularly after oligo- or asymptomatic SARS-CoV-2 infection, seroconversion is detected in an assay-dependent manner^6^. Moreover, several studies have reported a substantial decay of humoral immune responses and neutralizing capacity within 8 months and a rapid decline within the first 3 months pointing to a continuous loss of humoral immunity to the virus^7–10^.

Although studies have been performed to determine SARS-CoV-2 seroprevalences during the 1st pandemic wave in subpopulations^2,3^, so far few reliable data are available on the adult SARS-CoV-2 infection rate in Germany. A local seroprevalence study in a German hot-spot setting of a provincial town conducted after a superspreading event during carnival 2020 estimated an adult infection fatality rate (IFR) as low as 0.36% (95% CI: [0.29; 0.45]^2^. Another study conducted from April to June 2020 in the urban environment of Munich representative for individuals 14 years or older living in private households reported an infection fatality rate of 0.86% (95% CI: [0.67; 1.23]) if all deaths were counted, or 0.47% (95% CI: [0.36; 0.67]) if only 54% of deaths were counted assumed to occur in households^3^. Based on data from international meta-analyses^11–13^ with the assumption of an equal distribution of infections among all age groups the infection fatality rate in Germany has been estimated to be 1.14% (95% CI: [0.76; 1.51]) by German authorities^14^. Thus, apart from the reliability of the applied antibody test, estimates for infection rates can vary largely depending on the target population, sampling bias and period during which the study was conducted.

Here we performed a federal-state-wide cross-sectional seroprevalence study SaarCoPS representative for the adult population including elderly people and nursing home residents in the 1st pandemic wave in Germany. We selected one out of three commercial SARS-CoV-2 antibody assays with superior specificity and longitudinal assay performance to determine the SARS-CoV-2 seroprevalence in the study population, to estimate the infection rate, the underestimation ratio, and the infection fatality rate in a German federal state. Our results demonstrate that seroprevalence data are highly dependent on the particular assay technique used to determine SARS-CoV-2-specific antibodies, which in turn affects infection rate estmates. We estimate an adult infection rate of 1.02% (95% CI: [0.64; 1.44]), an underreporting rate of 2.68-fold (95% CI: [1.68; 3.79]) and infection fatality rates of 2.09% (95% CI: (1.48; 3.32]) or 0.36% (95% CI: [0.25; 0.59]) in all adults including elderly individuals, or adults younger than 70 years, respectively. These results provide a valuable baseline for future evaluation of pandemic dynamics including the impact of upcoming new viral variants, vaccination and public health measures on virus spread and human health.

## Methods

### Study design and ethical approval

The SaarCoPS study was conducted in the federal state of Saarland with an overall adult population of 845,000 inhabitants in six districts following GCP criteria. Saarland is located in southwestern Germany bordering the state of Rhineland-Palatinate and the countries France and Luxembourg. Study design, data management, access to data files and data protection issues were defined in a study protocol. The design and conduct of the study were supported by epidemiology and biometry institutions, e.g., the Saarland Cancer Registry at the Ministry of Health, Social Affairs, Women and Family and the Institute of Medical Biometry, Epidemiology and Medical Informatics at Saarland University Medical Center. On the basis of IFR data reported in a German study available at that time^2^ and COVID-19-related deaths in Saarland, a sample size of 2305 individuals was calculated, necessary to estimate an overall proportion of seropositive individuals of 4% with a relative precision of 0.2 (confidence level 95%). Overall, 10,000 inhabitants aged 18 years or older representative for the Saarland population with respect to age, sex and location were sampled from population registries and invited in two steps with an assumed participation rate of 30%. The Questor Pro software (Blubbsoft, Leipzig, Germany) was used for pseudonymization, generation of online questionnaires (covering questions, i.e. on age, sex, nursing or retirement home status), and data hosting. Serum samples and questionnaires were collected in 29 general practitioners‘ (GPs) offices distributed across the Saarland or mobile teams from July 22 to October 15, 2020. The study was approved by the Ethics Committee of the state of Saarland (Ärztekammer des Saarlandes, Saarbrücken, Germany) in accordance with the Declaration of Helsinki. Written informed consent was given by the study participants.

### Specimen collection and anti-SARS-CoV-2 antibody testing

In addition to sera from the study participants, serum or plasma samples from randomly selected 30 convalescent non-hospitalized individuals with previous PCR-confirmed asymptomatic or mild SARS-CoV-2 infection (mild fatigue or respiratory symptoms that did not require hospitalization, score 1–2 according to the contemporary WHO ordinal scale classification) were collected at the Institute of Virology, Saarland University Medical Center between April and October 2020 primarily from contact tracing performed as a public health service. The time interval between PCR-positivity and first blood sampling was 23.17 ± 4.81 days in samples where information on both exact time points were available (*n* = 18). Samples from hospitalized patients with COVID-19 disease were collected during the same time period as the samples from convalescent non-hospitalized individuals and tested (Supplementary Fig. 1) but deliberately excluded from this study to avoid a bias toward potentially stronger antibody responses in patients with more severe disease.

Serum or plasma samples from individuals with a pre-pandemic PCR-proven infection by endemic coronaviruses (OC43, NL63, HKU1 and 229E) were selected from the clinical sample repository of the Institute of Virology for retrospective testing of cross-reactivity in anti-SARS-CoV-2 antibody assays. All blood samples were investigated in three anti-SARS-CoV-2 antibody test systems (Table 1) suitable for the analysis of serum or plasma samples. Parallel testing of serum and plasma from the same patient yielded comparable results confirming another study using a different SARS-CoV-2 antibody assay^15^. The following assays were used.

**Table 1:** Anti-SARS-CoV-2 antibody assays.

Assay Name	Anti-SARS-CoV-2-ELISA	SARS-CoV-2 IgG	Elecsys Anti-SARS-CoV-2
Provider	Euroimmun	Abbott	Roche
Cat. number	EI 2606–9601 G	6R86-22	09203095190
Assay Principle	ELISA	CMIA^a^	ECLIA^b^
Antigen	SP S1 domain	Nucleocapsid protein (NCP), C-Terminus	Nucleocapsid protein (NCP)
Detected Antibody	IgG	IgG	Total Ig
Abbreviation	Euroimmun-IgG	Abbott-IgG	Roche-Ig
Cut-Off value	1.1	1.4	1.0
Sensitivity (%)	84.9 [81.5; 87.9]^c^	88.9 [85.8; 91.6]	90.3 [87.4; 92.8]
Specificity (%)	99.3 [98.29; 99.76]	99.4 [98.50; 99.84]	100.0 [99.46; 100]

^a^Chemoluminescence-microparticle immunoassay.

^b^Electrochemoluminescence immunoassay.

^c^95% confidence interval.

(1) A semiquantitative, automated Euroimmun assay (IgG: Cat# EI 2606–9601 G, IgA: Cat# EI 2606–9601 A, Lübeck, Germany) detecting SARS-CoV-2-specific IgG or IgA antibodies targeting the spike protein (S1 domain) and an Euroimmun Analyzer I that measures the OD of the samples. The assay is a classical ELISA, with stripes coated with SARS-CoV-2 antigen, that is incubated with samples and probed with enzyme-labeled anti-human IgG/IgA antibodies. Results are provided as OD and ratio of OD(Sample)/OD(calibrator). A threshold of 0.8 indicates borderline result, above a threshold of 1.1 a sample was considered as positive. (2) A two-step immunoassay for the qualitative detection of IgG antibodies directed against the nucleocapsid protein (NCP) of SARS-CoV-2 (Abbott, Cat# 6R86-22, Wiesbaden, Germany). This assay is based on the chemiluminescence-microparticle immunoassay (CMIA) technique. Briefly, sample, paramagnetic beads coated with SARS-CoV-2 antigen and diluent were incubated together. SARS-CoV-2-specific IgG antibodies bind to the antigen-coated beads. After washing, the acridinium-labeled anti-human IgG conjugate was added and incubated. After washing, pre-trigger and trigger solution were added and the resulting chemiluminescence reaction measured as relative light units. The amount of detectable IgG is directly proportional to the measured signal. Results are given as index (signal probe/signal calibrator) and considered positive above a threshold of 1.4. (3) The third assay is based on the electrochemiluminescence immunoassay (ECLIA) technique for the qualitative detection of antibodies directed against the NCP of SARS-CoV-2 (Elecsys Anti-SARS-CoV-2 assay, Roche Diagnostics GmbH, Cat# 09203095190, Frankfurt, Germany). Thus, unlike the above assays, the Roche-Ig test detects all immunoglobulins (Ig), and not solely IgG or IgA antibodies. This assay is based on a sandwich principle. Briefly, samples are sandwiched between biotinylated SARS-CoV-2-specific recombinant antigen and SARS-CoV-2-specific recombinant antigen labeled with a ruthenium complex. After addition of streptavidin-coated microparticles the complex is immobilized on the solid phase. The mixture is transferred into the measuring cells, where the magnetic beads are captured on the surface of the electrodes. After washing, a voltage pulse triggers chemiluminescence, which is measured by a photomultiplier. Results are provided as cut-off index (COI), and are considered as positive from a value of 1.0.

Data on sensitivity and specificity were kindly provided by the Paul-Ehrlich-Institute (PEI), a German federal institute, and respective test performance characteristics were further used for correction of seroprevalences. At PEI, 84% of the individuals tested had a very low COVID-19 symptom score of 1–2 (out of 7); specificity was tested on 676 pre-pandemic negative blood samples (100 serum, 576 citrated plasma samples). Part of these data were recently published^16^.

### Data processing, statistics and reproducibility

To estimate the seroprevalence in the general population, the observed seroprevalences were adjusted for age and sex using direct standardization (weights were derived from the population of the calendar year 2018, Supplementary Table 1). Confidence intervals (CI) were calculated using R version 4.0.3 as suggested by Waller and colleagues^17^. For sex and age strata 95% Blyth-Still-Casella CI were calculated using the rbscCI package of R^18^. Specific estimates were derived for the following ages: 18–44, 45–69, 18–69, ≥70 years). Adjusted estimates of seroprevalences were further corrected using test performance characteristics (sensitivity and specificity) of each individual test (data from PEI, Table 1) by applying following equation: (prevalence + specificity − 1)/(sensitivity + specificity − 1)^19^. The 95% confidence intervals for corrected seroprevalence, underestimation ratio and infection fatality rate were estimated using percentile bootstrap confidence intervals from 50,000 bootstrap samples^20^. For this purpose, the sensitivity and specificity were estimated from bootstrapped samples of the PEI data and the adjusted seroprevalence was estimated from bootstrapped samples of the SaarCoP study data. Subsequently, the corrected seroprevalence was estimated using the Rogan-Gladen estimator^19^.

Seroconversion occurs ~10–14 days after infection^21^. According to the Robert Koch-Institute, the median time from symptom to death was 11 days during the 1^st^ pandemic wave in Germany^20^, while multinational studies reported an interval of 16–18 days^22–24^. We therefore used both serological results and registered COVID-19 death numbers 14 days after the registered numbers of SARS-CoV-2 PCR-confirmed cases for both the estimation of infection and fatality rates. Case-fatality rate was calculated as the ratio of COVID-19 death cases (14 days after the PCR case reporting date) in relation to PCR-confirmed SARS-CoV-2-positive cases (Supplementary Table 2). Underestimation ratio is the underreporting rate calculated as the ratio of age- and sex-adjusted seroprevalences (reporting date October 15, 2020) that were corrected with respect to test performance characteristics, in relation to SARS-CoV-2 PCR-positive cases (reporting date October 1, 2020, Supplementary Table 3). Infection fatality rate (IFR) was calculated as the ratio of COVID-19 death cases (reporting date October 15, 2020, Supplementary Table 4) in relation to age- and sex-adjusted seroprevalences that were corrected with respect to test performance characteristics. Data were illustrated and statistical calculations performed with Graph Pad Prism 9 (Graph Pad Software, San Diego, USA) using the indicated tests. Significant differences were accepted if *p* ≤ 0.05. Test statistic (t) and degree of freedom (df) are indicated.

### Reporting summary

Further information on research design is available in the Nature Research Reporting Summary linked to this article.

## Results

### Choice and performance of antibody assays

**Fig. 1:**
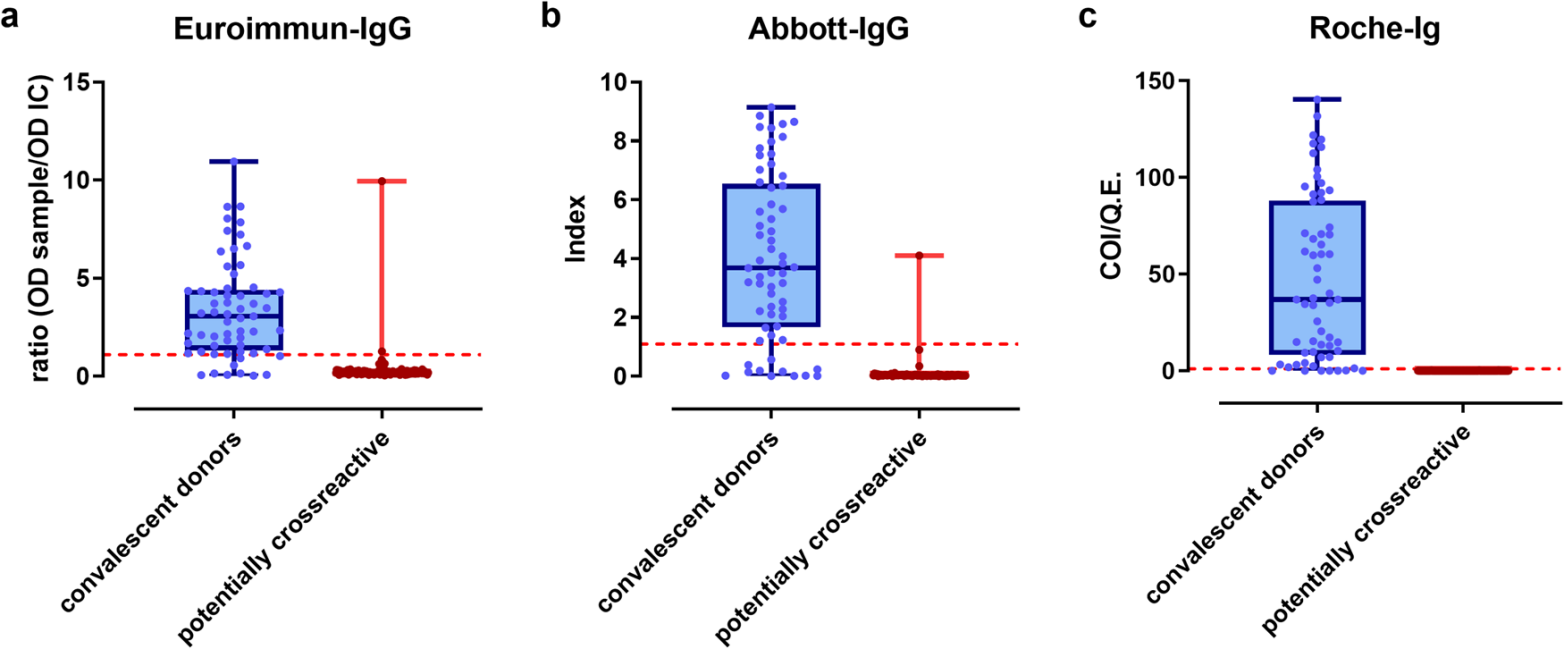
Assay performance data. Evaluation of the test performance using sera from convalescent donors after asymptomatic or mild infection, or pre-pandemic sera from donors after PCR-approved infections with endemic coronavirus strains to test potential cross-reactivities. Data are illustrated as box and whiskers blots showing minimum and maximum and all data points as OD ratio for Euroimmun-IgG (**a**), index for Abbott-IgG (**b**) and COI/Q.E. for Roche-Ig (**c**). Sample size for convalescent donors was 61, for potentially cross-reactive sera 128 (EI-IgG, Roche-Ig) and 78 (Abbott-IgG) (Table 2).

Since few data were available on the performance of SARS-CoV-2 antibody tests when we planned our study^6^, we compared three different test systems. The Euroimmun assay was included as one of the first commercial ELISAs available on the German market, and therefore widely used in other German seroepidemiological studies^2–4^. Assays from Abbott and Euroimmun measuring nucleocapsid-, or spike protein-directed IgG antibodies, respectively, had a lower test performance than the Roche assay detecting total antibodies against nucleocapsid protein. In our analyses, Roche-Ig displayed 100% specificity (95% CI: [97.02; 100.00]) with pre-pandemic sera from patients with previous PCR-approved infections with endemic coronavirus strains OC43, NL63, HKU1 or 229E, and a high sensitivity (90.16%, 95% CI: [79.81; 96.30]) for detection of SARS-CoV-2 antibodies in our sera from convalescent donors (Fig. 1, Table 2). Our results were confirmed by the German authorities in a parallel study (PEI, Table 1)^16^.

**Table 2:** Assay performance: calculation of the sensitivities with sera from convalescent donors and specificities based on the individual assay performances in A.

Convalescent donors				Potentially cross-reactive sera			
	EI-IgG	Abbott-IgG	Roche-Ig		EI-IgG	Abbott-IgG	Roche-Ig
*n*	61	61	61	*n*	128	78	122
Positive	52	49	55	Crossreaction	2	4	0
% Negative	14.75	19.67	9.84	%	1.67	1.69	0
Sensitivity (%)	85.25	80.33	90.16	Specificity (%)	98.33	98.31	100
CI (%)	73.83; 93.02	68.16; 89.04	79.81; 96.30	CI (%)	94.47; 99.81	87.39; 98.59	97.02; 100.00

Euroimmun-IgG = EI-IgG. 95% confidence intervals (CI %) were calculated according to Clopper and Pearson^34^.

### SARS-CoV-2 antibody assays strongly differ in longitudinal performance

**Fig. 2:**
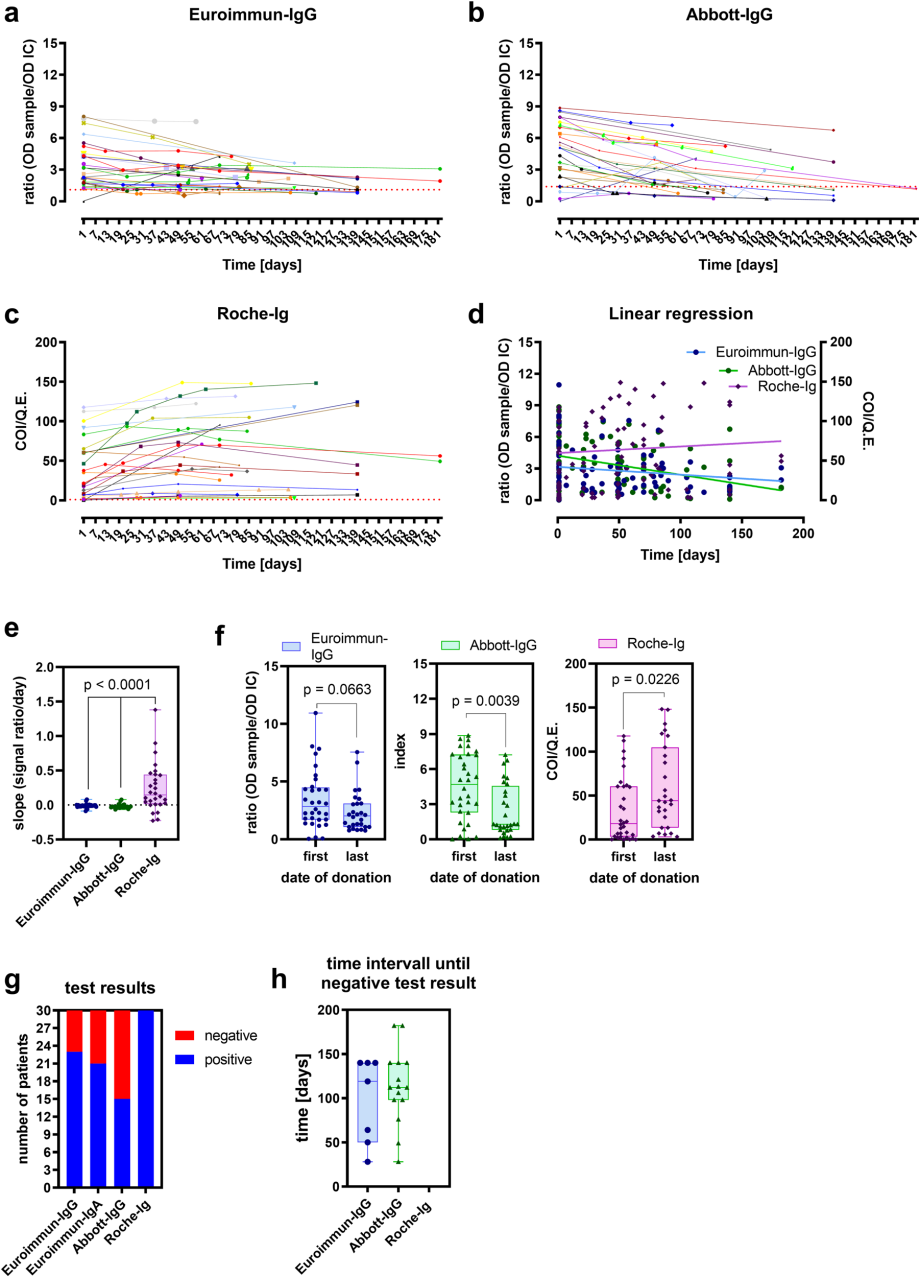
Differences in longitudinal performances of SARS-CoV-2 antibody test systems. **a**–**c** Time course of serological test results from the same 30 convalescent donors; identical sera were tested in three different assays (Euroimmun-IgG, Abbott-IgG and Roche-Ig). **d** Calculation of the linear regression overall data points of each individual assay results shown in **a**–**c**. **e** Calculation of the mean slope by simple linear regression analysis. **f** Comparison of test results of the first and last serum donation in the individual assays. **g** Number of positive and negative test results at the last individual date of blood donation. **h** Time interval after which a negative antibody test result was obtained from convalescent donors with previously positively tested sera. Data are illustrated as OD ratio for Euroimmun-IgG, index for Abbott-IgG and COI/Q.E. for Roche-Ig in **a**–**d** and **f**, slope (signal ratio/days) in **e**, numbers of patients in **g**, time (days) in **h** as box and whiskers blots showing all data points with minimum and maximum. Significances were calculated with one-way ANOVA with Tukey correction (t = 3.005, df = 110) in **e** and unpaired two-tailed *t*-test (t = 15.05, df = 58) in **f** and resulting *p*-values are depicted.

Antibody tests used for seroprevalence studies should ideally recognize seropositive individuals during the entire study period after seroconversion. To evaluate this longitudinal test performance, we collected consecutive sera from randomly selected 30 convalescent non-hospitalized individuals after mild SARS-CoV-2 infection from April to October 2020 and analyzed the identical sera in the three test systems (Fig. 2). During this time course, the antibody levels for Euroimmun-IgG and Abbott-IgG declined over time in the majority of cases (Fig. 2a, b, Supplementary Fig. 2). Subsequent linear regression analyses demonstrated opposing longitudinal trends of antibody levels measured with Euroimmun-IgG or Abbott-IgG in comparison to the Roche-Ig assay (Fig. 2d). While mean slopes of individual longitudinal curves were negative for Euroimmun-IgG and Abbott-IgG assays, a significantly different, positive slope (0.25 ± 0.35) was obtained with the Roche-Ig assay (Fig. 2e). Accordingly, in contrast to Euroimmun-IgG and Abbott-IgG results, antibody levels measured with the Roche-Ig assay were significantly higher at the last date of serum donation compared to the first date (Fig. 2f). Importantly, at the final date of serum donation, the Euroimmun-IgG test failed to detect antibodies in previously positive sera in 7 (23.3%) and the Abbott-IgG assay in 15 (50%) samples, respectively (Fig. 2g). The median time intervals to seroreversion were 97.29 ± 48.46 (95% CI: [52.47; 142.1]) days for Euroimmun-IgG or 113.1 ± 42.23 (95% CI: [89.75; 136.5]) days for Abbott-IgG, respectively. In strong contrast, no seroreversion was observed with the Roche-Ig assay during a period of more than 180 days (Fig. 2h).

These data demonstrate that anti-SARS-CoV-2 antibody test results can substantially differ from each other depending on the applied assay system. Due to its superior specificity and longitudinal test performance, the Roche-Ig assay appeared to be particularly suitable for use in SARS-CoV-2 seroprevalence studies.

### Case-fatality rate during the 1st pandemic wave in the German federal state Saarland

**Fig. 3:**
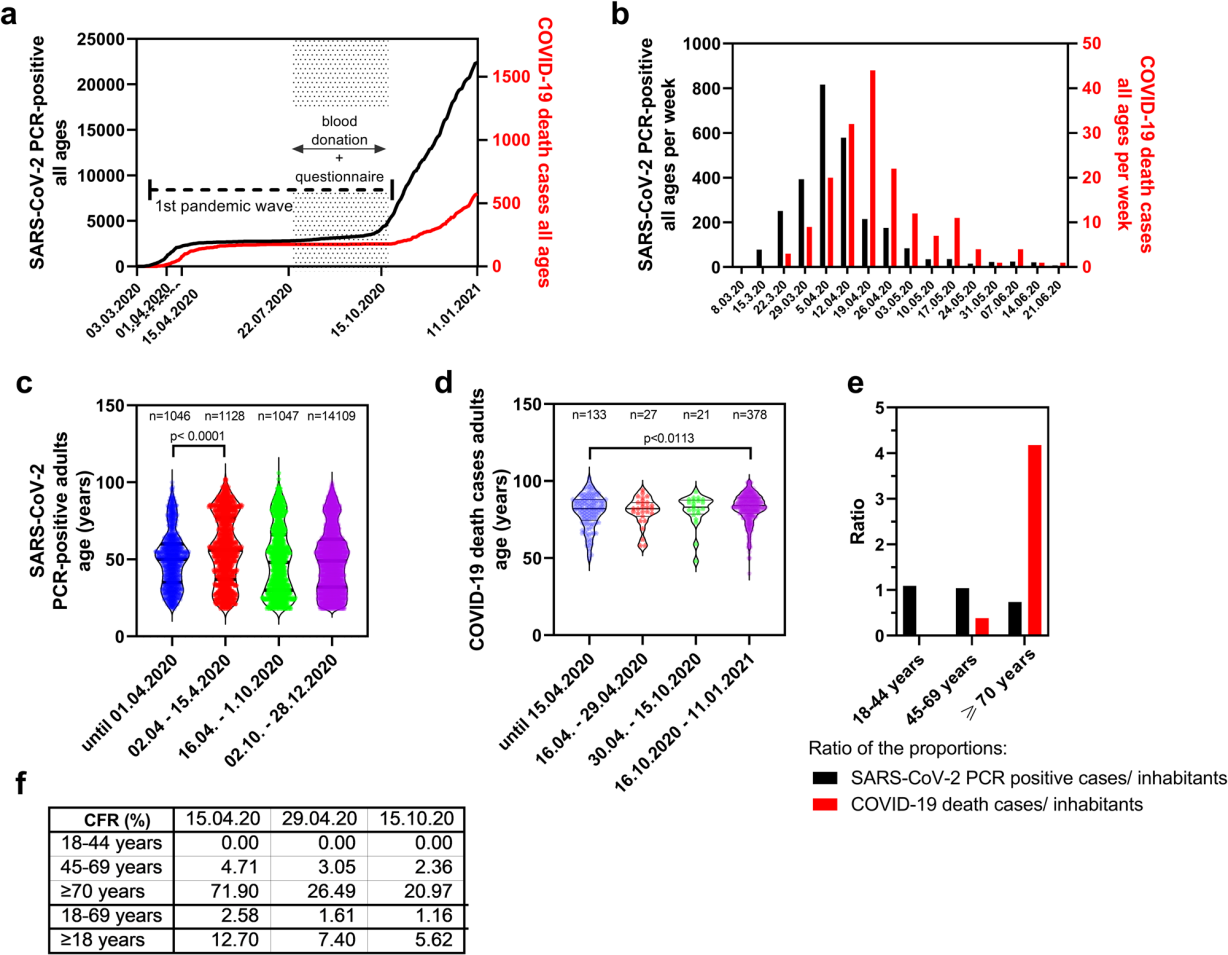
SARS-CoV-2 PCR-confirmed cases in Saarland: Time course, mean age and death cases. **a** Reported SARS-CoV-2 PCR-positive cumulative infections (black) and deaths (red) in Saarland from March 3, 2020 to January 11, 2021 (data source: Saarland Ministry of Health). First pandemic wave and time frame of the seroprevalence study (July 22 to October 15, 2020) are indicated. **b** SARS-CoV-2 PCR-positive cases (black) and COVID-19 death cases (red) in Saarland per calendar week. **c** Violin plot with median ages of PCR-positive cases during the indicated time intervals. (until 01.04.2020 *n* = 1046; 02.04.–15.04.2020 *n* = 1128; 16.04.–01.10.2020 *n* = 1047; 02.10.–28.12.2020 *n* = 14109). **d** Violin plot with median of death cases per age during the indicated time intervals (time frames shifted for 14 days compared to **b**. Until 15.04.2020 *n* = 133; 16.04.–29.04.2020 *n* = 27; 30.04.–15.10.2020 *n* = 21; 16.10.2020–11.01.2021 *n* = 378). Significances were calculated with unpaired two-sided *t*-test, *p*-values are depicted (B: t = 7.229, df = 2172; C: t = 2.543, df = 509). **e** Ratio of the proportion of SARS-CoV-2 PCR-confirmed cases by 01.04.2020 (black), or the proportion of COVID-19 death cases by 15.04.2020 (red) in the indicated age groups, relative to the proportion of Saarland inhabitants in the indicated age groups. **f** Time trends of case-fatality-rates (CFR) in Saarland at indicated time points for 18–44-, 45–69-year-old adults, elderly individuals ≥70 years, 18–69-year-old adults and all adults (≥18 years). Calculations are based on data in Supplementary Table 2.

In Germany the 1st pandemic wave with the subsequent low level plateau phase ended in mid of October 2020, when a second exponential increase was observed indicating the begin of the 2nd pandemic wave (Fig. 3a). Until mid of April 2020, a steep increase of infections and subsequent deaths (Fig. 3b) was noted in Saarland and an adult case-fatality rate (CFR) as high as 12.7% was calculated based on PCR-confirmed results (Fig. 3f, Supplementary Table 2). Local outbreaks in long-term care facilities and retirement homes contributed to this increase with about 9% of all PCR-confirmed infections and more than 50% of all deaths occurring in senior care homes in the Saarland until October 15, 2020 (data source: Saarland Ministry of Health). They largely concerned individuals aged 70 years or older leading to a significant transient shift of the median age (*p* < 0.0001) of PCR-confirmed SARS-CoV-2 infected individuals from 49.9 to 56.2 years as shown in violin plots (Fig. 3c). The CFR in age group ≥70 years was calculated 71.9%, in adults <70 years 2.58%. Between July and October 2020 cumulative SARS-CoV-2 infections and death cases reached a plateau in all affected age groups. The median age of death cases increased slightly from 80.2 to 80.9 years until October 15, 2020, and significantly increased further to 82.7 years (*p* < 0.0113) after the first wave (Fig. 3d). CFRs declined to 5.62% in adults of all age groups and to 1.16% in adults <70 years during the 1st pandemic wave (Fig. 3f, Supplementary Table 2).

Improvements in the healthcare system may have contributed to the reduction of the CFRs during time^25^. However, it was reasonable to assume that also underreporting of asymptomatic or oligosymptomatic SARS-CoV-2 infections not tested by PCR had largely veiled the actual infection and fatality rates during the 1st pandemic wave.

### Selection of a representative adult study population in the federal state Saarland

**Fig. 4:**
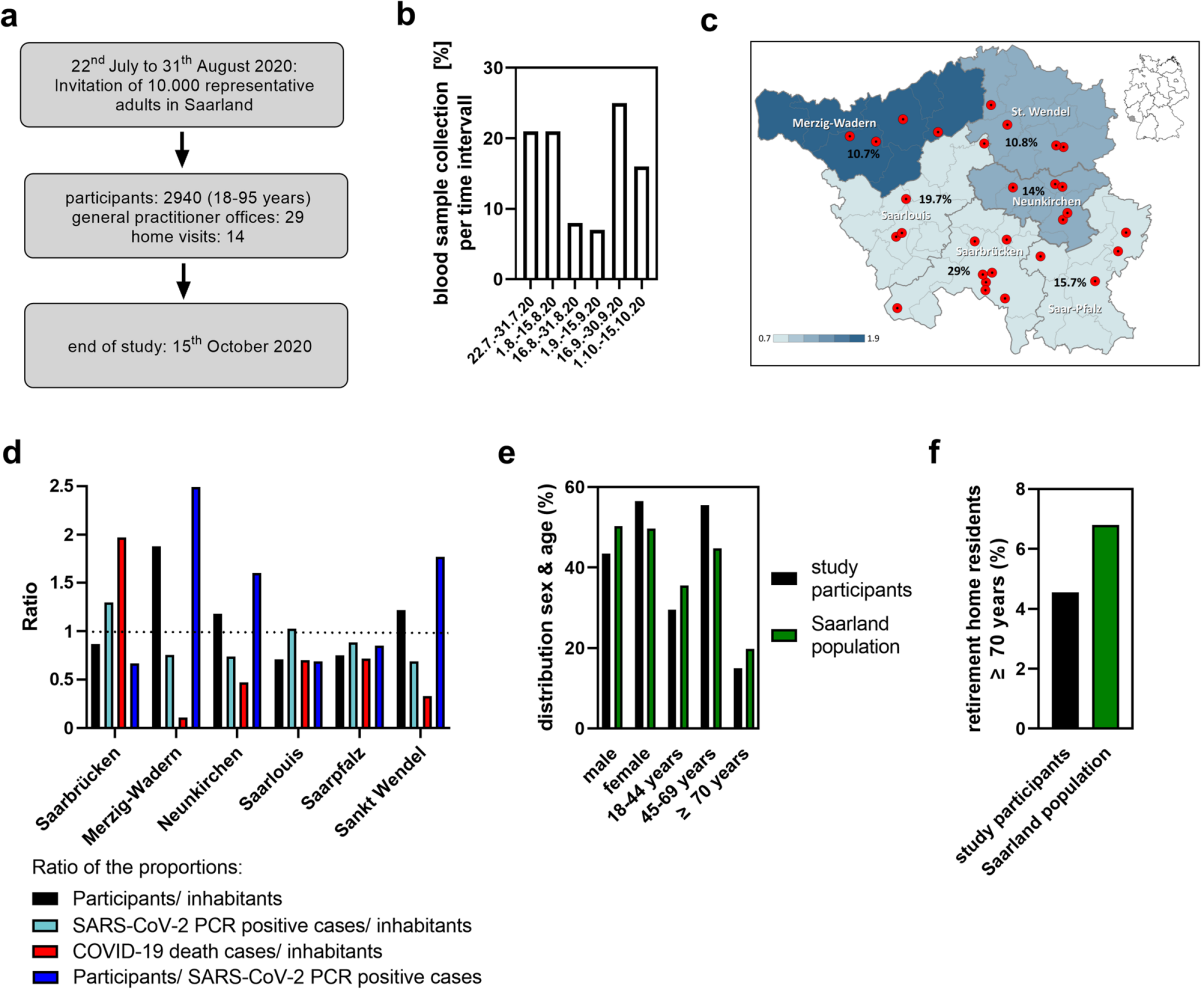
Study design and composition of the study population. **a** Time scale of study enrollment. **b** Percentages of blood samples from study participants collected during the indicated time frames of the study. **c** Numbers (%) indicate the proportion of study participants from respective administrative districts. The color graduation indicates the relative representation of the resident district population within the study population from 0.7 to 1.9 in steps of 0.2. Administrative boundaries from © GeoBasis-DE / BKG (2020), Data licence Germany—attribution—version 2.0. **d** Shown is the quotient of the relative proportions of study participants per district (black), SARS-CoV-2 PCR-positive cases per district (light blue), COVID-19 death cases per district (red), respectively, and the relative proportion of residents in that district, or the quotient of the relative proportion of study participants per district (blue) and the relative proportion of SARS-CoV-2 PCR-positive cases per district by October 15, 2020. **e** Sex and age characteristics of study participants in relation to the resident population (Supplementary Table 1). **f** Proportion of individuals aged 70 years or older living in nursing or retirement homes in the study sample and the overall population^25^.

The seroprevalence study was conducted from July 22 (~2 months after infections and 1 month after deaths had again declined, see Fig. 3b) to October the 15th, 2020, after which an exponential increase indicated the start of the 2nd pandemic wave (Fig. 3a). The intention was to cover the entire 1st pandemic wave, including the subsequent plateau phase as depicted in Fig. 4a. Overall, 10,000 individuals representative for the adult Saarland population with respect to age, sex and location were invited and 2940 participated in the study (participation rate 29.4%). Until August the 15th, 2020, about 40%, until September the 15th 55% and until October the 15th 100% of participants donated blood (Fig. 4b). The relative composition (%) and representation (colour code) of the six administrative districts is indicated in Fig. 4c. Easy access to the study was ensured by 29 GPs offices distributed across the Saarland (Fig. 4c). Mobile teams provided home visits for elderly, handicapped or otherwise immobile individuals, of which 14 participants made use. Figure 4d (black bars) shows that relative participation varied between 0.71 (Saarlouis) and 1.88 (Merzig-Wadern) among the administrative districts. However, it also shows that the relative SARS-CoV-2 PCR-positivity among the six districts varied only between 0.89 (Neunkirchen and Merzig-Wadern) and 1.12 (Saarbrücken), indicating that infections were relatively evenly distributed in Saarland and that there were no hotspots of infection in Saarland (Fig. 4d, light blue bars). Our study also revealed that there were larger differences in SARS-CoV-2-related deaths between the districts (Fig. 4d, red bars). Particularly in Merzig-Wadern, which was relatively overrepresented in our study, only few deaths were observed. However, this district represents only 10.5% of the Saarland population. Overall, 56.5% of the participants were females (Fig. 4e). Of the participants, 29.5% were <45, 55.5% were 45–69 and 15% were 70 years or older. In Saarland, about 6.8% of individuals aged 70 years or older are residents of a retirement or nursing home^26^. Notably, 4.55% of our study participants in this age group resided in respective homes indicating that this important elderly subpopulation was well reflected in our study (Fig. 4f).

### Adult SARS-CoV-2 seroprevalences in 1st pandemic wave

Sera from all study participants were analyzed with the three different anti-SARS-CoV-2 antibody assays. Fifty four out of 2940 study participants tested positive at least in one of these assays, 33 individuals in the Abbott-IgG test, 28 in Euroimmun-IgG test and 27 in the Roche-Ig test. Thirteen sera showed a positive reactivity in all three assays (Supplementary Table 5). Notably, none of the participants‘ sera reacted only in Abbott-IgG and Euroimmun-IgG but not in the Roche-Ig test.

**Fig. 5:**
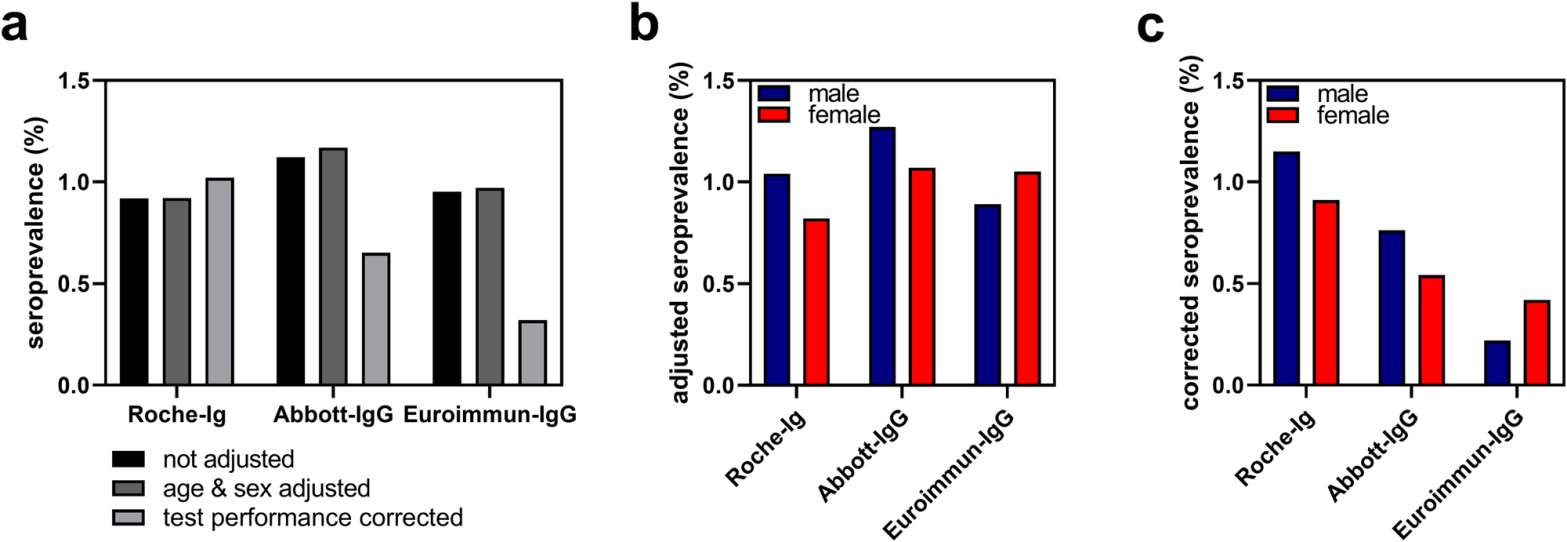
Comparison of unadjusted, age- and sex-adjusted seroprevalences as well as corrected seroprevalences. **a** Comparison of results obtained with Roche-Ig, Abbott-IgG and Euroimmun-IgG assays. **b** Sex- and age-adjusted seroprevalence data of males and females. **c** Seroprevalences of males and females corrected for sensitivity and specificity. For correction, validation data from PEI were used (Table 1)^16^. 95% confidence intervals are shown in Supplementary Table 6.

Crude seroprevalences were calculated for each antibody assay (Fig. 5a). After adjustment for age and sex (Suppl. Table 6) seroprevalences were estimated 1.17% (95% CI: [0.87; 1.78]) for Abbott-IgG, 0.97% [0.71; 1.55] for Euroimmun-IgG and 0.92% (95% CI: [0.67; 1.48]) for Roche-Ig. The respective values for males and females varied depending on the assay used (Fig. 5b, Supplementary Table 6). In a third step, we corrected the data with respect to test performances previously determined by PEI according to Table 1. This resulted only in a slight increase of the calculated seroprevalence measured with Roche-Ig assay, which was 1.02% (95% CI: [0.64; 1.44]) for all adults, 1.15% (95% CI: [0.54; 1.87]) in males and 0.91% (95% CI: [0.48; 1.39]) in females (Fig. 5a, c, Supplementary Table 6). However, for both, Abbott-IgG and Euroimmun-IgG, assays this correction led to dramatic changes, particularly for the Euroimmun-IgG test that displays lowest sensitivity and specificity, resulting in a corrected seroprevalence as low as 0.32% (Fig. 5a, c), and no reasonable confidence intervals could be calculated (Supplementary Table 6). These results highlighted the importance of a high test performance of antibody assays used in seroprevalence studies, particularly when seroprevalences are low.

### Estimation of the underreporting ratio and infection fatality rate (IFR) in adults

**Fig. 6:**
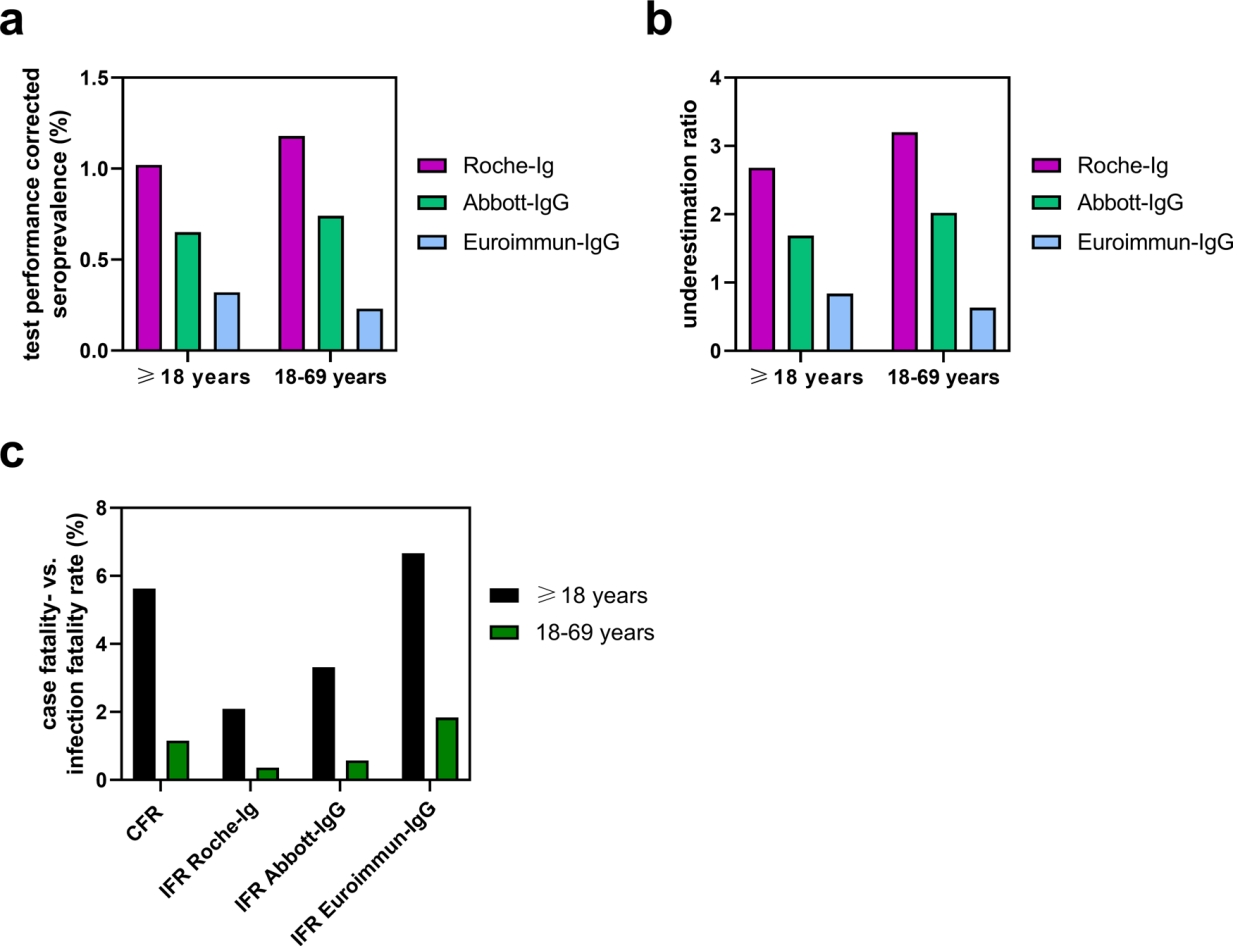
Assay-dependent seroprevalence, underestimation ratio and estimation of IFR. **a** Test performance-corrected seroprevalence rates on October 15, 2020. **b** Underestimation ratio as calculated by corrected seroprevalence in relation to the number of reported PCR-positive cases in Saarland (valuation date October the 1st, 2020). **c** Infection fatality rates as calculated from the CFRs and respective underreporting rates on October 15, 2020, the end of the 1st pandemic wave. Data in the subfigures **a**–**c** were obtained with three different antibody tests systems, either in all adults or in adults younger than 70 years, respectively, as indicated. Respective 95% confidence intervals are shown in Supplementary Table 6.

Assuming that seroconversion requires around 14 days after infection in the 1st pandemic wave, the underreporting ratio of SARS-CoV-2 infections over all adults was calculated from performance-corrected seroprevalence data on October 15, 2020 (Fig. 6a) and the rate of PCR-confirmed cases in Saarland on October the 1st, 2020 (0.38%) (data from the Saarland Ministry of Health, Social Affairs, Women and the Family) as 2.68-fold (95% CI: [1.68; 3.79]) for Roche-Ig. Again, results obtained with the other test systems differed strongly, 1.69 for Abbott-IgG and 0.84 for Euroimmun-IgG, and reasonable 95% confidence intervals could not be calculated (Fig. 6b, Supplementary Table 6). Data obtained with the Roche-Ig assay indicated that the estimated number of infected adults in Saarland was much higher (more than 8600 cases) than the reported PCR-confirmed cases on October 1, 2020 (3221 cases). Comparison of the three antibody test systems showed that this underestimation ratio could not be unravelled with test systems of inferior sensitivity, specificity and longitudinal performance, resulting in a much lower (Abbott-IgG) or no underreporting rate (Euroimmun-IgG). Using the Roche-Ig test we estimated an infection fatality rate (IFR) at the end of the 1st pandemic wave in Saarland of 2.09% (95% CI: [1.48; 3.32]). For Abbott-IgG or for Euroimmun-IgG the estimates were 3.32% or 6.66%, respectively, the latter IFR being even higher than the calculated CFR of 5.62%, and reasonable 95% confidence intervals could not be calculated (Fig. 6c, Supplementary Table 6). Thus, the assay performance is a very critical parameter affecting the accuracy of IFR calculation.

Infection-related death rates in nursing or retirement homes can largely drive the overall SARS-CoV-2 IFR in adult populations^14^. We therefore additionally performed our analyses for adults <70 years, assumed to live in private households rather than in nursing homes. In this adult subpopulation, a CFR of 1.16% was calculated until October 15, 2020. The age- and sex-adjusted seroprevalence was 1.06% (95% CI: [0.75; 1.68]), for males 1.07% (95% CI: [0.60; 2.07]) and for females 1.06% (95% CI: [0.65; 1.77]) with the Roche-Ig assay (Suppl. Table 6). After correction for test performance we estimated a seroprevalence of 1.18% (95% CI: [0.72; 1.68]) (Fig. 6a), an underestimation ratio of 3.20-fold (95% CI: [1.96; 4.60]) (Fig. 6b, Supplementary Tables 3 and 6) and an IFR of 0.36% (95% CI: [0.25; 0.59]) (Fig. 6c, Supplementary Tables 4 and 6) in the Saarland adult population <70 years, which was substantially lower than in adults of all ages including the elderly nursing-home-based population.

## Discussion

In this study we have estimated the SARS-CoV-2 infection and infection fatality rates during the 1st pandemic wave from a German federal-state-wide cross-sectional seroprevalence study representative for the adult resident population. With 1.02% (95% CI: [0.64; 1.44]) seropositivity, the estimated infection rate was 2.68-fold (95% CI: [1.68; 3.79]) higher than registered PCR-confirmed cases. We estimated an infection fatality rate of 2.09% (95% CI: [1.48; 3.32]) over all adults, and in adults younger than 70 years of age an infection rate of 1.18% (95% CI: [0.72; 1.68]) and an infection fatality rate of 0.36% (95% CI: [0.25; 0.59]).

### Strengths of our study

Strengths of our study are the (1) study population which is representative for the general population as it also included nursing home residents, (2) the time frame of the study covering the complete time period before the 2nd pandemic wave, (3) the accounting for the delays of seroconversion and death after infection for calculations of infection and fatality rates, (4) the reduction of assay-dependent uncertainties by choosing an antibody test system with maximal specificity, and, importantly, (5) limiting seroreversion effects by using an antibody test system with high longitudinal performance.

### Importance of the representativeness of the studied population

Our study was designed to randomly select a representative registry-based study population. Age- and sex adjustments of calculated seroprevalence rates did not grossly differ from crude values indicating that the study participants were indeed representative for the adult population of the Saarland. The slight over-representation of females and the middle ages were also reported in other studies^2^. IFR estimates can vary widely depending on the investigated population as shown in German and international studies (Supplementary Table 7). A study in a German population 14 years and older living in private households in the city of Munich^3^, or an early hot-spot study in the adult population of the small town Gangelt^2^ (with only seven death cases) estimated infection fatality rates of 0.36–0.67% or 0.29–0.45% (95% confidence intervals), respectively. In contrast, in another German hot-spot study in Tirschenreuth using the Roche-Ig and an in-house ELISA, an IFR of 2.49% (95% CI: [2.06; 3.02]) of those aged 14 years and older was estimated, where 56 of 129 deaths (43.4%) occurred in senior care homes until May 11, 2020^27^. This was similar to the IFR of 2.09% (95% CI: [1.48; 3.32]) in adults during the 1st pandemic wave estimated in the present SaarCoPS study, where more than 50% of all deaths occurred in senior care homes until October 15, 2020. Thus, SaarCoPS covered the critical time frame during the 1st pandemic wave, during which the attack rate in nursing homes and the IFR were extremely high due to a lack of appropriate prevention strategies and hygiene measures. Our data suggest a dramatic increase of the IFR from age 70 on (Supplementary Fig. 3B) albeit the small numbers of participants with SARS-CoV-2 infection did not allow to investigate statistical significances of fatal cases with respect to age. Our results also matched with the estimates from a study using an age-specific modelling framework based on a log-linear pattern. From 10 representative antibody studies the overall IFR was estimated to be 0.78–1.79% (95% prediction interval range) in high-income countries with higher proportions of elderly individuals^12^. The importance of the age composition of the study population and the critical contribution of nursing homes residents to age-specific COVID-19 mortality is supported by other national and international studies^11,14,27^. In particular, Levin et al. describe in their meta-analysis an exponential relationship between age and IFR and that IFR depends essentially on how much vulnerable groups were affected by SARS-CoV-2 infections during the observation period^11^. In our study, stationary as well as mobile blood-collecting teams also ensured that elderly people, including those living in nursing or retirement homes, which have a particular impact on the adult fatality rate, were well represented in our study. In contrast to our and the Tirschenreuth study^27^, the Munich study focusing on private households^3^ and the German hot-spot study conducted after a carnival superspreading event^2^, however, may have missed nursing home data, which might have led to an underestimation of the true infection fatality rate. In fact, the exclusion of individuals aged 70 or older from our study population resulted in an infection fatality rate of 0.36% (95% CI: [0.25; 0.59]) (Fig. 6a) that is comparable to the results of the aforementioned German studies. Other German studies may be even less representative for the elderly population due to a focus on special cohorts with substantial age-restrictions, such as blood donors^2–5,28–30^, resulting in lower IFRs^3,28^. Interestingly, in the age group of 70 years or older, the underestimation ratio was estimated only 1.16-fold in our study (Supplementary Fig. 3C). This observation may be explained by the fact that a SARS-CoV-2 infection more frequently causes disease symptoms in the elderly and by the extensive PCR-based screening of nursing and retirement homes in Saarland using a pooling strategy^31^.

### Importance of longitudinal antibody test performance for seroprevalence calculation and IFR estimation

A very important factor creating differences between seroepidemiological studies comes from assay-dependent uncertainties^1–3^. For our study, we evaluated three different antibody test systems and chose the one with highest specificity and highest longitudinal performance for our calculations. Our data show, that the anti-SARS-CoV-2 Roche-Ig assay can detect antibody responses for at least 180 days after SARS-CoV-2 infection, whereas substantial seroreversions were observed with the Abbott-IgG and the Euroimmun-IgG antibody assays.

Compared to the Roche-Ig assay, the Abbott-IgG assay led to a 36.91% and the Euroimmun-IgG assay to a 68.55% lower estimated infection rate in adults after correction for test performance and to lower underestimation ratios (Fig. 6a, b, Supplementary Table 6). Compared to the Roche-Ig assay, this resulted in estimation of 1.59-fold or 3.18-fold higher IFRs (Fig. 6c) obtained with Abbott-IgG or Euroimmun-IgG assays, respectively, for which reasonable confidence intervals could not be calculated.

Both the Abbott-IgG and Roche-Ig assays detect antibodies to the SARS-CoV-2 nucleocapsid protein. The Abbott-IgG assay shows a substantial decay rate. An important finding of this study, however, is that the Roche-Ig test shows virtually no decline. It can therefore be assumed that the viral target protein is not the determining factor for the differences in the longitudinal test performance. A similar decay as for the Abbott assay was reported for an in-house anti-nucleocapsid IgG assay^8^. A major difference between these two anti-nucleocapsid IgG or Euroimmune anti-spike IgG assay, and the Roche-Ig assay is their assay design. While the isotype-specific IgG antibody assays use an indirect test format, the Roche-Ig assay detects total antibodies in a sandwich test design. Detection of “total antibodies” (independent of Ig class) compared to “IgG only” maximizes sensitivity, which explains the higher baseline sensitivity, but more importantly, the sandwich assay design can exploit higher antibody affinities, resulting in higher detection duration.

Our data were confirmed by a study of the German federal Paul-Ehrlich-Institute PEI, which demonstrated a high longitudinal performance of the Roche-Ig assay with a larger cohort and seropositivity up to 430 days after symptom onset, while isotype-specific IgG assays showed a lower detection duration^16^. Notably, they reported that the sensitivity of the Roche-Ig assay increases from 84% 30 days after symptom onset to 98.8–100% within 60–300 days after symptom onset in individuals with mild infections (severity scores 1–3). Thus, even in this patient collective, antibody levels are maintained and remain detectable for months in total antibody assays, which agrees well with our observations. They explained this interesting phenomenon by an increase of the antibody avidity and affinity maturation of the antibody repertoire over time^16^.

To account for potential limitations of our own test performance data due to small sample size and incomplete data on time frames after PCR-positivity, we used the preliminary test performance data of the PEI for corrections, which, however, corresponded well with our data obtained from individuals with asymptomatic or mild infections. The time interval between the begin of the pandemics in Saarland (03.03.2020) and the end of blood donation (15.10.2020) for the seroprevalence study was 226 days, and thus well within the time frame of 300 days, in which the Roche-Ig test sensitivity increased to 98.8–100%^16^. The use of a test sensitivity of 90.3% for corrections of seroprevalence calculations within the present study was therefore considered rather conservative.

Although the nucleocapsid-based Roche-Ig assay has been superior over time to the other assays for seroepidemiologic studies, the correlation with viral neutralization may be reflected more by the antibody response to the SARS-CoV-2 spike protein-based assays. Here, the use of the full S-trimer as antigen for a SARS-CoV-2 IgG assay was shown to give higher test sensitivity than an S1 domain-based assay (both from the same manufacturer Euroimmun)^32^. These assays may therefore be more appropriate to answer questions concerning humoral individual or population immunity to SARS-CoV-2, boosting of humoral responses after reinfection, or immune responses after vaccination.

## Conclusions and outlook

From our study we conclude that seroepidemiological investigations benefit from assays with the highest possible sensitivity, specificity and longitudinal test performance to achieve reliable data, particularly when seroprevalence is low, as in the 1st pandemic wave in Germany. We conclude that 2.68-fold more individuals were infected with SARS-CoV-2 during this period indicating that a high number of infections was missed to effectively contain viral spread. Our study also unravelled a realistic picture on a high adult infection fatality rate of 2.09% (95% CI: [1.48; 3.32]) during the 1st pandemic wave, since elderly and nursing home residents were adequately represented in our study.

In summary, our study results provide a valuable basis to evaluate the future effects of the pandemic development particularly in comparison to neighbouring countries, such as Luxembourg or France, including the impact of SARS-CoV-2 variants of concern, which have ingressed during the 2nd pandemic wave and started to dominate in March 2021, and fatality rates after availability of SARS-CoV-2 vaccines.

A second generation of recently established multiplex antibody assays such as MuliCoV-Ab^33^ with high test performance, might be of great value to further distinguish between vaccine responses, SARS-CoV-2 and endemic human coronaviruses infection, and potentially also new coronavirus variants^14^.

## Supplementary information

Reporting Summary

Supplementary Material

Supplementary Data

Description of Additional Supplementary Files

## Data Availability

All data that support the findings of this study are available within the article or its Supplementary Information. Supporting data are available in the Supplementary Data file, which contains the raw data.
