# Supplementary material for: German federal-state-wide seroprevalence study of 1st SARS-CoV-2 pandemic wave shows importance of long-term antibody test performance
Source: Commun Med (Lond). 2022 May 18;2:52. doi: 10.1038/s43856-022-00100-z (PMC9117207; doi:10.1038/s43856-022-00100-z)
Supplement: Supplementary file 1 — Reporting Summary [file 43856_2022_100_MOESM1_ESM.pdf]

nature research

Corresponding author(s): Sigrun Smola, sigrun.smola@uks.eu

Last updated by author(s): 26.01.2022

Reporting Summary

Nature Research wishes to improve the reproducibility of the work that we publish. This form provides structure for consistency and transparency in reporting. For further information on Nature Research policies, see our Editorial Policies and the Editorial Policy Checklist.

Statistics

For all statistical analyses, confirm that the following items are present in the figure legend, table legend, main text, or Methods section.

n/a	Confirmed	
☐	☒	The exact sample size (n) for each experimental group/condition, given as a discrete number and unit of measurement
☐	☒	A statement on whether measurements were taken from distinct samples or whether the same sample was measured repeatedly
☐	☒	The statistical test(s) used AND whether they are one- or two-sided<br>*Only common tests should be described solely by name; describe more complex techniques in the Methods section.*
☒	☐	A description of all covariates tested
☐	☒	A description of any assumptions or corrections, such as tests of normality and adjustment for multiple comparisons
☐	☒	A full description of the statistical parameters including central tendency (e.g. means) or other basic estimates (e.g. regression coefficient) AND variation (e.g. standard deviation) or associated estimates of uncertainty (e.g. confidence intervals)
☐	☒	For null hypothesis testing, the test statistic (e.g. F, t, r) with confidence intervals, effect sizes, degrees of freedom and P value noted<br>*Give P values as exact values whenever suitable.*
☒	☐	For Bayesian analysis, information on the choice of priors and Markov chain Monte Carlo settings
☒	☐	For hierarchical and complex designs, identification of the appropriate level for tests and full reporting of outcomes
☒	☐	Estimates of effect sizes (e.g. Cohen's d, Pearson's r), indicating how they were calculated

Our web collection on statistics for biologists contains articles on many of the points above.

Software and code

Policy information about availability of computer code

Data collection	Questor Pro Software, Blubbsoft company, Microsoft Excel 2016
Data analysis	R Version 4.0.3, Microsoft Excel 2016, GraphPad Prism 9

For manuscripts utilizing custom algorithms or software that are central to the research but not yet described in published literature, software must be made available to editors and reviewers. We strongly encourage code deposition in a community repository (e.g. GitHub). See the Nature Research guidelines for submitting code & software for further information.

Data

Policy information about availability of data

All manuscripts must include a data availability statement. This statement should provide the following information, where applicable:

- Accession codes, unique identifiers, or web links for publicly available datasets
- A list of figures that have associated raw data
- A description of any restrictions on data availability

All data that support the findings of this study are available within the article or its supplementary materials.

Field-specific reporting

Please select the one below that is the best fit for your research. If you are not sure, read the appropriate sections before making your selection.

☒ Life sciences ☐ Behavioural & social sciences ☐ Ecological, evolutionary & environmental sciences

For a reference copy of the document with all sections, see nature.com/documents/nr-reporting-summary-flat.pdf

Life sciences study design

All studies must disclose on these points even when the disclosure is negative.

Sample size	A sample size of 10000 individuals was calculated based on the assumption that 30% of invited individuals will attend.
Data exclusions	An available case analysis was performed: only persons that agreed to participate, send blood samples and answered the questionnaire were included.
Replication	The individual data points of each biological replicate is indicated.
Randomization	Randomization of subjects were not required due to the study design (cross-sectional study) and the underlying scientific questions
Blinding	Blinding of investigators were not required due to the study design (cross-sectional study) and the underlying scientific questions

Reporting for specific materials, systems and methods

We require information from authors about some types of materials, experimental systems and methods used in many studies. Here, indicate whether each material, system or method listed is relevant to your study. If you are not sure if a list item applies to your research, read the appropriate section before selecting a response.

Materials & experimental systems

n/a	Involved in the study	
☐	☒	Antibodies
☒	☐	Eukaryotic cell lines
☒	☐	Palaeontology and archaeology
☒	☐	Animals and other organisms
☐	☒	Human research participants
☒	☐	Clinical data
☒	☐	Dual use research of concern

Methods

n/a	Involved in the study	
☒	☐	ChIP-seq
☒	☐	Flow cytometry
☒	☐	MRI-based neuroimaging

Antibodies

Antibodies used	Euroimmun assay (IgG: Cat# EI 2606-9601 G, IgA: Cat# EI 2606-9601 A, Lübeck, Germany); Abbott assay (Cat# 6R86-22, Wiesbaden, Germany); Elecsys Anti-SARS-CoV-2 assay (Roche Diagnostics GmbH, Cat# 09203095190, Frankfurt, Germany).
Validation	The three antibody test systems are commercially available and established CE-marked IVD test systems validated by the individual companies.

Human research participants

Policy information about studies involving human research participants

Population characteristics	2940 out of 10000 invited individuals participated in the study, 56·5% of the participants were females. Of the participants, 29·5% were < 45, 55·5% were 45-69, and 15% were 70 years or older, 4·55% of study participants 70 years and older lived in retirement or nursing homes
Recruitment	10000 inhabitants aged 18 years or older were sampled from population registries and invited by regular mail. All documents were enclosed (invitation letter, declaration of consent, questionnaire, list of participating physicians). Questionnaire could be answered online as well. Serum samples and questionnaires were collected in 29 general practitioners` (GPs) offices distributed across the Saarland from July 22nd to October 15th 2020. Mobile teams made home visits or visited retirement homes to reach immobile/older people.
Ethics oversight	The study was approved by the Ethics Committee of the state of Saarland (Ärztekammer des Saarlandes, Saarbrücken, Germany) in accordance with the Declaration of Helsinki. Written informed consent was given by the study participants.

Note that full information on the approval of the study protocol must also be provided in the manuscript.
