# Supplementary material for: German federal-state-wide seroprevalence study of 1st SARS-CoV-2 pandemic wave shows importance of long-term antibody test performance
Source: Commun Med (Lond). 2022 May 18;2:52. doi: 10.1038/s43856-022-00100-z (PMC9117207; doi:10.1038/s43856-022-00100-z)
Supplement: Supplementary file 2 — Supplementary Material [file 43856_2022_100_MOESM2_ESM.pdf]

Supplementary Material

Supplementary Figure 1: Test performance comparing sera or plasma from convalescent non-hospitalized, or hospitalized COVID-19 patients, potentially crossreactive and pre-pandemic control sera or plasma.

Sera or plasma of individual donors were tested for SARS-CoV-2 specific antibodies using the Eurimmun-IgG, the Abbott-IgG and Roche-Ig assays. Data are illustrated as OD ratio for Euroimmun-IgG, index for Abbott-IgG and COI/Q.E. for Roche-Ig.

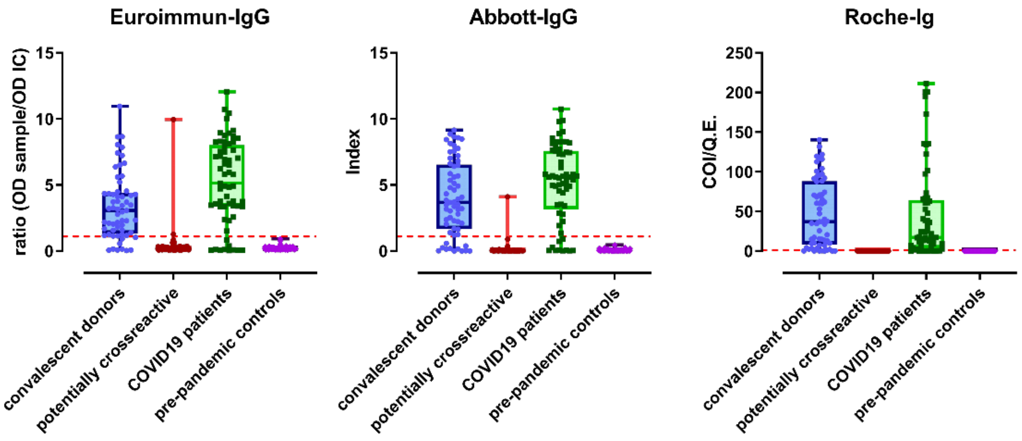

Supplementary Figure 2: Assay-dependent longitudinal serological results of individual donors

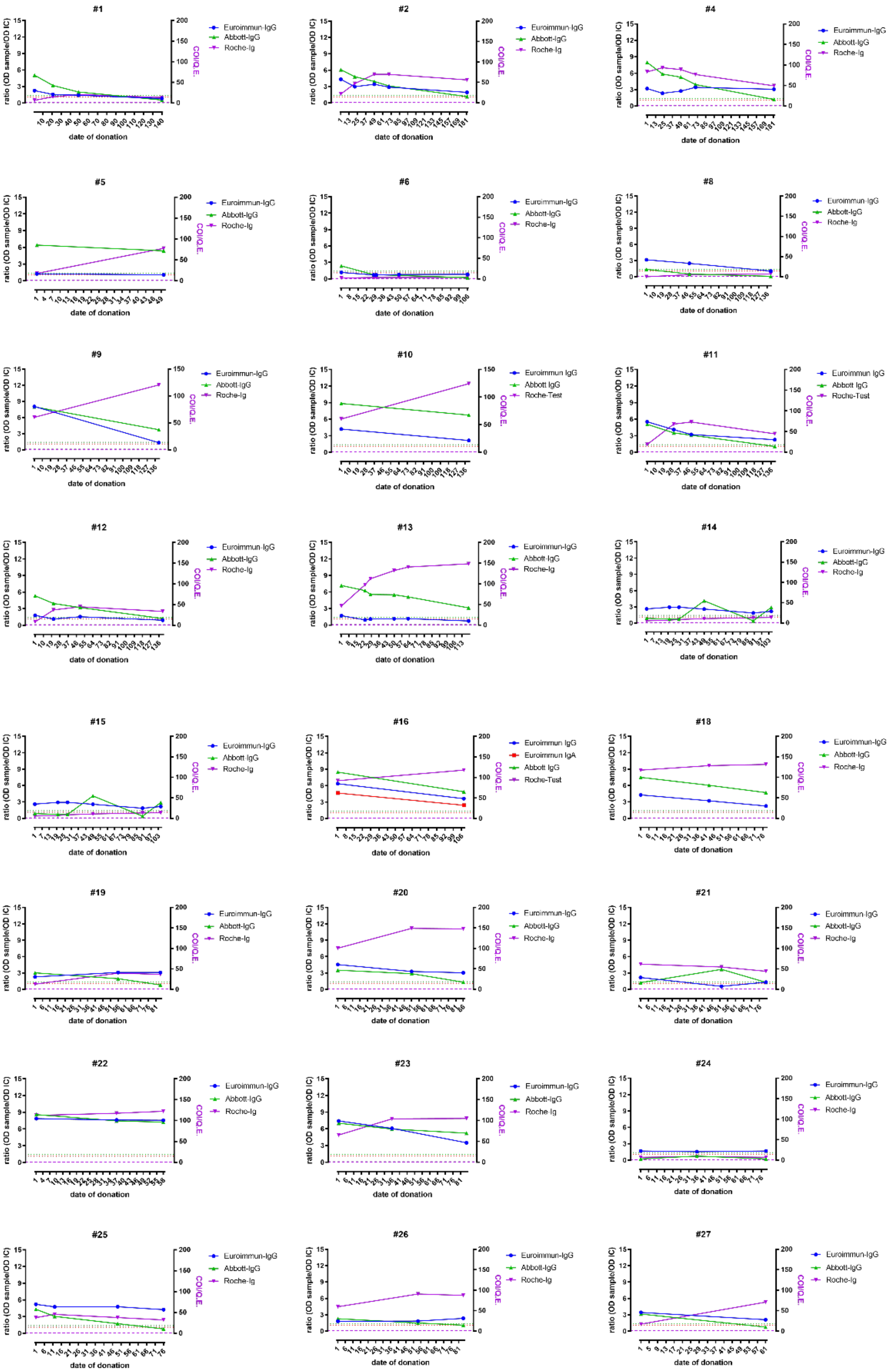

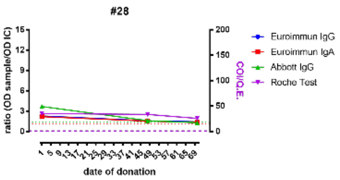
#28
ratio (OD sample/OD IC)
COI/U.E.
date of donation
Euroimmun IgG
Euroimmun IgA
Abbott IgG
Roche Test

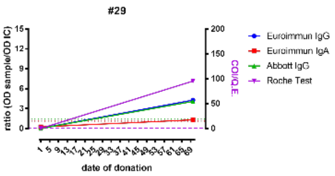
#29
ratio (OD sample/OD IC)
COI/U.E.
date of donation
Euroimmun IgG
Euroimmun IgA
Abbott IgG
Roche Test

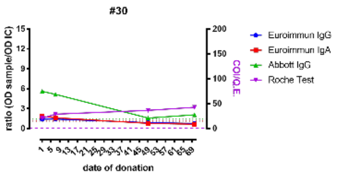
#30
ratio (OD sample/OD IC)
COI/U.E.
date of donation
Euroimmun IgG
Euroimmun IgA
Abbott IgG
Roche Test

Supplementary Table 1: Distribution of age- and sex in the study sample and general population of Saarland in 2018

	**Total (n=2940)**			**male (n=1278)**			**female (n=1662)**		
	18-44 years	45-69 years	≥ 70 years	18-44 years	45-69 years	≥ 70 years	18-44 years	45-69 years	≥ 70 years
**Study population**	868	1632	440	328	714	236	540	918	204
**% of total**	30	56	15	26	56	19	33	55	12
**Saarland population in 2018 (*based on census in 2011*)**	299782	377860	167231	154703	186473	69659	145079	191387	97572
**Weighting (≥18 years)***	0.35	0.45	0.20	0.38	0.45	0.17	0.33	0.44	0.22
**Weighting (18-69 years)****	0.44	0.56	-	0.45	0.55	-	0.43	0.57	-

*Weighting of the stratum to calculate the positive rates for SARS-CoV-2 antibodies for Supplementary Table 3

**Weighting of the stratum to calculate the positive rates for SARS-CoV-2 antibodies for the ages 18-69

Supplementary Table 2: CFR was calculated by dividing cumulative COVID-19 death numbers (as of the reporting date 14 days after the PCR case reporting date) by cumulative SARS-CoV-2 positive cases in the specified age group.

Age [years]	CFR 15.4.20 COVID-19 death cases 15.4.20/ SARS-CoV-2 positive 01.4.20	CFR 29.4.20 COVID-19 death cases 29.4.20/ SARS-CoV-2 positive 15.4.20	CFR 15.10.20 COVID-19 death cases 15.10.20/ SARS-CoV-2 positive 01.10.20
**18-44**	0/405	0/787	0/1269
**45-69**	23/488	27/885	29/1227
**≥ 70**	110/153	133/502	152/725
**18-69**	23/893	27/1672	29/2496
**≥ 18**	133/1046	160/2174	181/3221

Supplementary Table 3: The underestimation ratio was calculated by dividing the corrected seroprevalence (not rounded) on 15.10.20 by the SARS-CoV-2 positivity rate as of 01.10.20 in Saarland in the indicated age group.

Age [years]	Underestimation ratio (Roche-Ig) 15.10.20 corrected seroprevalence / PCR positivity rate	Underestimation ratio (Abbott-IgG) 15.10.20 corrected seroprevalence / PCR positivity rate	Underestimation ratio (Euroimmun-IgG) 15.10.20 corrected seroprevalence / PCR positivity rate
**18-69**	1.18/(2496/677,642) = 3.20	0.74/(2496/677,642) = 2.02	0.23/(2496/677,642) = 0.63
**≥ 18**	1.02/(3221/844,873) = 2.68	0.65/(3221/844,873) = 1.69	0.32/(3221/844,873) = 0.84

Supplementary Table 4: The IFR was calculated by dividing cumulative COVID-19 death numbers on 15.10.20 by the corrected seroprevalence (not rounded) on 15.10.20 multiplied by Saarland residents in the specified age group.

Age [years]	IFR (Roche-Ig) 15.10.20 COVID-19 death cases / (corrected seroprevalence * inhabitants in age group)	IFR (Abbott-IgG) 15.10.20 COVID-19 death cases / (corrected seroprevalence * inhabitants in age group)	IFR (Euroimmun-IgG) 15.10.20 COVID-19 death cases / (corrected seroprevalence * inhabitants in age group)
**18-69**	29/(1.18*677,642) = 0.36	29/(0.74*677,642) = 0.58	29/(0.23*677,642) = 1.84
**≥ 18**	181/(1.02*844,873)= 2.09	181/(0.65*844,873) = 3.32	181/(0.32*844,873) = 6.66

Supplementary Table 5: Numbers of individuals tested positive in at least one SARS-CoV-2 antibody test and total number of positive test results

Test positive in	individuals tested positive, n=54	18-44 years	45-69 years	≥70 years	male	female
**Abbott-IgG only**	14	3	9	2	6	8
**Euroimmun-IgG only**	13	3	6	4	6	7
**Roche-Ig only**	6	0	6	0	4	2
**Abbott-IgG & Roche-Ig**	6	4	2	0	3	3
**Euroimmun-IgG & Roche-Ig**	2	1	1	0	0	2
**Abbott-IgG, Euroimmun-IgG & Roche-Ig**	13	7	4	2	5	8
	total number of positive test results, n=88	18-44 years	45-69 years	≥70 years	male	female
**Abbott-IgG**	33	14	15	4	14	19
**Euroimmun-IgG**	28	11	11	6	11	17
**Roche-Ig**	27	12	13	2	12	15

Supplementary Table 6: Age- and sex- adjusted seroprevalences [95% confidence interval] in Saarland until October 15th 2020 obtained with three different tests (Roche-Ig, Abbott-IgG, Euroimmun-IgG) for all sexes, males or females. Correction of adjusted positivity rates according to [1] using data from the Paul-Ehrlich-Institute (Table 1).

	Roche-Ig	Abbott-IgG	Euroimmun-IgG
Age- and sex-adjusted			
≥18 years	0.92 [0.67; 1.48]	1.17 [0.87; 1.78]	0.97 [0.71; 1.55]
18-69 years	1.06 [0.75; 1.68]	1.26 [0.90; 1.91]	0.90 [0.62; 1.47]
male			
≥18 years	1.04 [0.62; 1.94]	1.27 [0.79; 2.24]	0.89 [0.53; 1.74]
18-69 years	1.07 [0.60; 2.07]	1.27 [0.73; 2.34]	0.72 [0.36; 1.60]
female			
≥18 years	0.82 [0.50; 1.49]	1.07 [0.71; 1.82]	1.05 [0.67; 1.85]
18-69 years	1.06 [0.65; 1.77]	1.24 [0.79; 2.00]	1.07 [0.64; 1.81]
corrected (sensitivity/specificity)*			
≥18 years	1.02 [0.64; 1.44]	0.65 [-0.20; 1.41]	0.32 [-0.66; 1.10]
18-69 years	1.18 [0.72; 1.68]	0.74 [-0.13; 1.56]	0.23 [-0.76; 1.02]
male			
≥18 years	1.15 [0.54; 1.87]	0.76 [-0.23; 1.78]	0.22 [-0.89; 1.14]
18- 69 years	1.19 [0.48; 2.01]	0.76 [-0.30; 1.89]	0.03 [-1.06; 0.96]
female			
≥18 years	0.91 [0.48; 1.39]	0.54 [-0.34; 1.38]	0.42 [-0.65; 1.33]
18-69 years	1.17 [0.61; 1.80]	0.73 [-0.21; 1.62]	0.44 [-0.64; 1.37]
underestimation ratio			
≥18 years	2.68 [1.68; 3.79]	1.69 [-0.49; 3.74]	0.84 [-1.74; 2.89]
18-69 years	3.20 [1.96; 4.60]	2.02 [-0.35; 4.24]	0.63 [-2.06; 2.79]
infection fatality rate			
≥18 years	2.09 [1.48; 3.32]	3.32 [-17.85; 26.16]	6.66 [-54.98; 60.53]
18-69 years	0.36 [0.25; 0.59]	0.58 [-2.38; 4.18]	1.84 [12.84; 13.30]

*** with respect to data from the Paul-Ehrlich-Institute**

Supplementary Table 7: Literature estimates on IFRs in various populations.

Location	Assay	IFR	Reference
**Germany**	10 different lateral flow assays (LFA), in-house ELISA	1.14% (95% CI:[0.76%; 1.51%])	2
**Germany, Munich**	Euroimmun-IgG, Roche-Ig	0.47% (95% CI:[0.36%; 0.67%])	3
**Germany, Gangelt**	Euroimmun-IgG	0.36% (95% CI:[0.29%; 0.45%])	4
**Denmark**	Livzon Diagnostics IgM/IgG-LFA	0.089% (95% CI: [0.072%; 0.211%])	5
**Switzerland, Geneva**	-	0.64% (95% CI:[0.38%; 0.98%])	6
**Spain**	Abbott-IgG	0.8% (95% CI:[0.8%; 0.9%])	7
**USA, New York City**	-	1.39% (95% CI:[1.04%; 1.77%])	8
**Germany (Kupferzell)**	Euroimmun-IgG	n.a.	2
**Germany (Munich)**	Roche-Ig	0.86 (95% CI:[0.67 – 1.23])	2
**Germany (Tirschenreuth)**	Roche-Ig, Shenzhen YHLO-IgG, in-house ELISA	2.5 % (95% CI:[2.06 – 3.02])	2
**Germany (Reutlingen)**	Euroimmun-IgG	1.3 (95% CI:[1.0 – 1.7])	2
**Germany (Aachen)**	Euroimmun-IgG	1.0 (95% CI:[0.7 – 1.3])	2
**Germany (Osnabrück)**	Euroimmun-IgG	1.6 (95%CI:[1.2-2.1])	2
**High-income countries**	-	1.15% (95% CI:[0.78%; 1.79%])	9

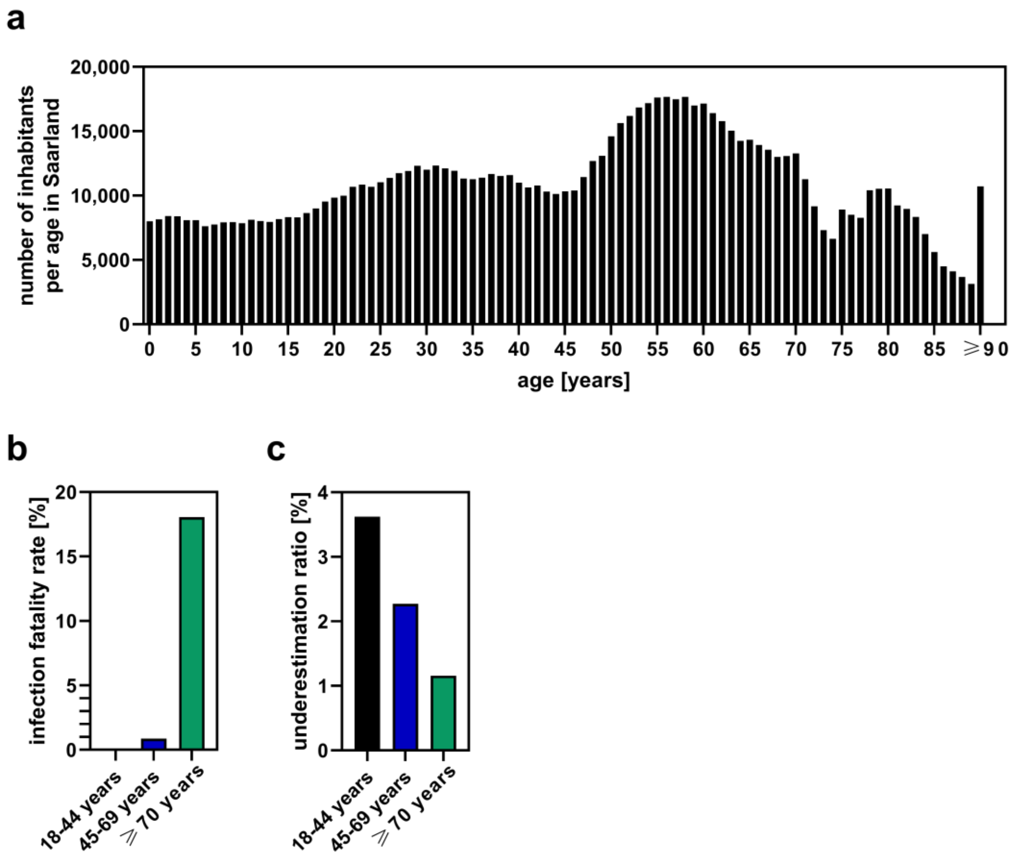

Supplementary Figure 3: a) Age distribution of Saarland inhabitants based on consensus 2011 b) Infection fatality rate and c) underestimation ratio (on 15th October 2020) of study participants were calculated for three different age groups. Calculations are based on test performance-corrected seroprevalence data only.

References Supplementary Material:

1. Santos-Hövener C, et al. Serology- and PCR-based cumulative incidence of SARS-CoV-2 infection in adults in a successfully contained early hotspot (CoMoLo study), Germany, May to June 2020. *Euro Surveill*. 25:2001752. 10.2807/1560-7917.ES.2020.25.47.2001752 (2020).

2. Neuhauser H, et al. Seroepidemiologische Studien zu SARS-CoV-2 in Stichproben der Allgemeinbevölkerung und bei Blutspenderinnen und Blutspendern in Deutschland – Ergebnisse bis August 2021. *Epid Bull*. 37:3 -12. 10.25646/8999 (2021). DOI 10.25646/9159

3. Pritsch M, et al. Prevalence and Risk Factors of Infection in the Representative COVID-19 Cohort Munich. *Int J Environ Res Public Health*. 30;18(7):3572. 10.3390/ijerph18073572. (2021).

4. Streeck H, et al. Infection fatality rate of SARS-CoV2 in a super-spreading event in Germany. *Nat Commun*. 11:5829. 10.1038/s41467-020-19509-y (2020).

5. Erikstrup C, et al. Estimation of SARS-CoV-2 infection fatality rate by real-time antibody screening of blood donors. *Clin Infect Dis.* 72:249-253. 10.1093/cid/ciaa849 (2021).

6. Perez-Saez, J. et al. Serocov-POP Study Group. Serology-informed estimates of SARS-CoV-2 infection fatality risk in Geneva, Switzerland. *Lancet Infect. Dis.*, 21, 69–70. 10.1016/S1473-3099(20)30584-3 (2021).

7. Pastor-Barriuso R, et al. ENE-COVID Study Group. Infection fatality risk for SARS-CoV-2 in community dwelling population of Spain: nationwide seroepidemiological study. *BMJ*. 371:m4509. 10.1136/bmj.m4509 (2020).

8. Yang W, et al. Estimating the infection-fatality risk of SARS-CoV-2 in New York City during the spring 2020 pandemic wave: a model-based analysis. *Lancet Infect Dis.*;21:203-212. 10.1016/S1473-3099(20)30769-6 (2021).

9. N. Brazeau, et al., "Report 34: COVID-19 infection fatality ratio: estimates from seroprevalence" (https://www.imperial.ac.uk/mrc-global-infectious-disease-analysis/covid-19/report-34-ifr/, 2020).
