# Supplementary material for: German federal-state-wide seroprevalence study of 1st SARS-CoV-2 pandemic wave shows importance of long-term antibody test performance
Source: Commun Med (Lond). 2022 May 18;2:52. doi: 10.1038/s43856-022-00100-z (PMC9117207; doi:10.1038/s43856-022-00100-z)
Supplement: Supplementary file 4 — Description of Additional Supplementary Files [file 43856_2022_100_MOESM4_ESM.pdf]

Description of Additional Supplementary Files

File Name: Supplementary Data

Description: Raw data file
